# Manual and Device-Assisted Hamstring Autograft Tensioning Yield Similar Outcomes following ACL Reconstruction

**DOI:** 10.3390/jcm12144623

**Published:** 2023-07-11

**Authors:** Andreas Piskopakis, Trifon Totlis, Vlasios Achlatis, Frantzeska Zampeli, Jim Dimitris Georgoulis, Michael Hantes, Nikolaos Piskopakis, Marios Vekris

**Affiliations:** 1 Department of Orthopaedics and Traumatology, KAT General Hospital, 14561 Kifissia, Greece; piskopakis@gmail.com (A.P.); fzampeli@gmail.com (F.Z.); md.piskopakis@gmail.com (N.P.); 2 Department of Orthopaedics and Sports Injuries, Medical Center of Athens, 15125 Marousi, Greece; 3 Thessaloniki Minimally Invasive Surgery (TheMIS) Orthopaedic Center, St. Luke’s Hospital, 55236 Thessaloniki, Greece; 4 Department of Anatomy and Surgical Anatomy, Faculty of Health Sciences, School of Medicine, Aristotle University of Thessaloniki, 54124 Thessaloniki, Greece; vlach899@gmail.com; 5 First Department of Orthopaedics, School of Medicine, National and Kapodistrian University of Athens, 10679 Athens, Greece; jim.georgoulis@gmail.com; 6 Department of Orthopaedic Surgery and Musculoskeletal Trauma, University Hospital of Larissa, 41334 Larissa, Greece; hantesmi@otenet.gr; 7 Department of Orthopaedic Surgery, School of Medicine, University of Ioannina, 45110 Ioannina, Greece; mdvekris@cc.uoi.gr

**Keywords:** ACL reconstruction, hamstring autograft, graft tensioning, device-assisted, clinical outcomes

## Abstract

The optimal initial graft tension during ACL reconstruction is still a matter of debate. Manual tension is commonly applied to the graft during tibial fixation. However, this has been associated with a greater graft failure rate than that associated with device-assisted tensioning. This study aims to compare the clinical outcomes between the application of manual tension and the use of the ConMed Linvatec SE™ Graft Tensioning System during graft fixation while performing anatomic single-bundle ACL reconstruction. Methods: A prospective comparative study was conducted between September 2015 and May 2017. Sixty-four patients (mean age 29.3 years, range 14–45) with isolated ACL injuries (and who would be subjected to ACL reconstruction with a quadruple hamstring tendon graft) were divided into two groups. In Group A (*n* = 29), common tension was applied manually to both grafts. In Group B (*n* = 35), specific tension was applied to the grafts with the use of a tensioner device (ConMed Linvatec SE™ (Stress Equalization) Graft Tensioning System). A total of 60 N was applied to the semitendinosus, and 40 N was applied to the gracilis. Clinical outcomes were assessed at 6, 12, and 24 months. Results: There were no significant differences between the baseline demographic and clinical data among the patients of the two groups (all *p* > 0.05). The patients were followed up for a minimum of 24 months (mean ± SD). There were no significant differences in the side-to-side anterior knee laxity, the IKDC, the Lysholm Knee, and the Tegner Activity Scale scores for up to 24 months after operation. The pivot shift test was negative in all cases, and no graft failure was reported at a 2-year follow-up. Conclusion: No significant differences were found with respect to postoperative anterior knee laxity, clinical outcomes, activity level, and patient satisfaction between the application of manual tension and the use of the graft-tensioning system during tibial fixation while performing anatomic single-bundle ACL reconstruction with a quadruple hamstring tendon graft. Further high-quality clinical studies are required to elucidate whether device-assisted tension is superior to manual tension.

## 1. Introduction

Anterior cruciate ligament (ACL) reconstruction with a patellar tendon or hamstring autograft is currently the most common surgical technique for managing ACL ruptures. ACL reconstruction techniques using autografts have shown satisfactory results. Implantation techniques, graft fixation, and tensioning, along with postoperative rehabilitation, have demonstrated significant improvement [1,2]. Despite the excellent clinical outcomes following ACL reconstruction, there are still several unanswered questions concerning the surgical technique.

An important variable affecting the outcome of ACL reconstruction is the in vivo tensioning of the graft at the time of fixation [3,4]. The importance of graft tensioning lies in its direct impact on the knee’s biomechanics and overall joint stability. The optimal initial graft tension during ACL reconstruction is still a matter of debate. Several studies support the application of a low level of initial graft tension, aiming to decrease the stresses within the graft, avoid the over-constraining of the knee, and eliminate the frictional force of articular surfaces [5,6,7]. On the other hand, low graft tension may sacrifice overall stability, thereby increasing anterior knee laxity, increasing the risk of re-injury, and compromising the clinical outcome [8]. Moreover, excessive tension can lead to graft failure, a limited range of motion, and potential complications, such as joint stiffness and arthrofibrosis [6].

Currently, the most common method for applying graft tension is manual tensioning, where a maximum one-handed pull on the graft is applied during tibial fixation, with an estimated tension of 41–60 N. Manual tensioning is an important step in the surgical procedure to ensure that the reconstructed ACL graft is appropriately tensioned. It involves applying a specific amount of tension to the graft to mimic the natural tension and length of the original ACL. The goal is to achieve optimal graft tension that allows for proper knee stability and a functional range of motion. Manual tension is the preferred tensioning method for 80% of Australian surgeons because of its easiness [9]. However, the disadvantages of manual tension include significant tension variation depending on the surgeons’ tractive power and skill, increased difficulty of the control of graft tension leading to an unknown level of initial tension, and suture loosening or breakage during knot tying. Moreover, the tension may drop after tibial fixation due to load relaxation of the femur–graft–tibia complex. Another option is the use of a tensioning device, where a mean tension of 81.85 N is applied to the graft using the device [9]. Device-assisted tensioning can help overcome some of the challenges associated with manual tensioning. These specialized devices have the theoretical advantage of achieving more precise and reproducible graft tension, allowing for controlled, objective, and standardized tensioning and reducing the risk of over- or under-tensioning the graft [10].

Several factors influence the graft-tensioning process. The choice of graft can impact the tensioning requirements. Different grafts (e.g., patellar and hamstring) have varying biomechanical properties and degrees of elasticity, thus affecting the ideal tensioning range [11]. Individual patient characteristics, such as age, activity level, knee laxity, and preoperative instability, should be considered when determining the appropriate level of graft tension. Younger and more active patients may require higher tension to withstand demanding physical activities [12]. Moreover, surgical factors, such as tunnel placement, graft fixation methods, and tunnel convergence angles, can influence graft tensioning. Finally, progressive rehabilitation exercises and physical therapy help optimize graft healing and promote appropriate graft tension [4].

While manual tensioning is simple, it causes a greater degree of variability in the amount of tension applied compared to device-assisted tensioning [13]. Previous clinical and cadaveric studies reported no postoperative differences in anterior tibial translation or clinical results among patients for whom device-assisted tensioning was compared to manual tensioning applied to hamstring autografts [10,14,15,16]. Other authors found that manual tensioning was associated with a higher graft failure rate than device-assisted tensioning [17].

The value of this study is that it offers information and data to assess whether the use of graft-tensioning systems provides any additional benefit for the patients in comparison to the application of manual tension during ACL reconstruction. Specifically, the aim of this study is to compare the clinical outcomes between the application of manual tension and the use of the ConMed Linvatec SE™ Graft Tensioning System during graft fixation while performing anatomic single-bundle ACL reconstruction with a quadruple hamstring tendon graft. The current study hypothesizes that applying a tensioning device may be associated with greater postoperative knee stability and better functional outcomes compared to applying manual tension.

## 2. Materials and Methods

A prospective, comparative study was conducted at our institution between September 2015 and May 2017. The study was approved by the corresponding institutional review board before initiation. All patients provided informed consent after a full explanation of the study protocol. Every participant of the study was skeletally mature and had been suffering from unilateral ACL deficiency at the time of surgery. The diagnosis of ACL tear was based on patients’ history, clinical examination, and magnetic resonance imaging (MRI) of the knee. Exclusion criteria were meniscal injuries, bony avulsions, multi-ligamentous injuries, chondral lesions, and osteoarthritis. Moreover, patients that had undergone any form of previous knee operation were excluded from the study.

Patients’ demographic data, such as gender, age at the time of operation, height, and weight, were recorded. Bone mass indices (BMIs) were also calculated. The time interval between injury and operation and the width of the femoral and tibial tunnels were also recorded. Sixty-four patients with isolated ACL injuries who were to be subjected to ACL reconstruction with a quadruple hamstring tendon graft were divided into two groups. In Group A (*n* = 29), common tension was applied manually to both grafts. In Group B (*n* = 35), a specific level of tension was applied to the grafts with the use of a tensioner device [ConMed Linvatec SE™ (Stress Equalization) Graft Tensioning System]. A total of 60 N was applied to the semitendinosus, and 40 N was applied to the gracilis tendon.

### 2.1. Surgical Technique

All subjects underwent an ACL reconstruction with a quadruple hamstring autograft performed by the first author. Each patient was positioned on the operating table under general anesthesia. The injured knee was prepared and draped in a sterile manner to minimize the risk of infection. The semitendinosus-gracilis tendons were initially harvested through a 4 cm incision over the ipsilateral pes anserinus. The width of the graft was measured. After diagnostic arthroscopy, any remaining torn ACL fibers were debrided to create a clean environment for the new graft. A femoral tunnel was created through the anteromedial arthroscopic portal with an outside–in technique. A tibial tunnel was created with a tibial guide placed in the anatomical position. The hamstring autograft was passed from the tibial tunnel to the external aperture of the femoral tunnel. It was fixed to the femur using a cortical suspension device (XO Button (ConMed Linvatec, Largo, FL, USA)). The angle of tibial fixation was 15°. The knee’s range of motion was determined. At this stage, the graft was tensioned via repeated cycling (20 cycles). A wire was passed through the tibial tunnel beside the graft. In Group A, graft tensioning was achieved through free one-hand pull force. In Group B, the graft was fixed using the ConMed Linvatec SE™ Graft Tensioning System, applying 60 N to the semitendinosus and 40 N to the gracilis. A tibial interference screw was placed centrally by separating the bundles of the graft and placing the bundles on the entire surface of the bone tunnel. The manual tension applied by the first author, using a dynamometer, was measured for 5 patients, and mean tension was calculated at 55.3 N.

The knee joint was thoroughly irrigated to remove any debris or blood. The incisions were closed with staples, and sterile dressings were applied. Patients were placed in a hinged knee brace for postoperative protection and stability. Postoperatively, all patients followed the same rehabilitation program. No running was allowed until four months after surgery. Return to full sports activity was generally permitted at nine months.

### 2.2. Assessment of Clinical Outcomes and Postoperative Knee Stability

The first author conducted the patients’ evaluations. A KT-1000 arthrometer (MEDmetric, San Diego, CA, USA) was used to measure anterior tibial translation at maximum manual pull, which is expressed as the difference between the injured and uninjured knee in millimeters (mm). The anterior tibial translation was measured preoperatively, intraoperatively under general anesthesia, and postoperatively at 0, 3, and 6 months following ACL reconstruction. The International Knee Documentation Committee Subjective Knee Form (IKDC), the Lysholm Knee Score, and the Tegner Activity Scale questionnaires (preoperatively, at 6, 12, and 24 months) were used to assess functional outcomes [18,19]. All patients attended the final follow-up (2 years).

### 2.3. Statistical Analysis

The values of the variables are presented using the number of participants (N), the mean values (M), and the standard deviations (SD). Frequencies (n) and the corresponding percentages (%) were used for categorical variables. The normality of distribution of the measurements was assessed using the Kolmogorov–Smirnov test. Comparisons of the variables at any given time between the two groups were performed using the *t*-test for independent samples or the Mann–Whitney test in case the data did not follow a normal distribution. All statistical analyses were performed with the statistical package SPSS, version 17.00 (SPSS Inc., Chicago, IL, USA). All tests were two-sided. A *p*-value of <0.05 was set as the level defining a statistically significant difference.

## 3. Results

The sample size was initially 111 patients subjected to ACL reconstruction. Preoperatively, 30 patients were excluded due to meniscal injuries, and 2 patients were excluded due to collateral ligament injuries or chondral lesions. After intraoperative inspection, 14 more patients were excluded due to meniscal injuries, and 1 patient was rejected due to a chondral lesion. Finally, 64 patients were included in the study.

The study cohort consisted of 64 patients (50 men-14 women), with a mean age of 29.3 years (range 14–45 years). The mean weight was 72.4 kg (range 61–88 kg), the mean height was 173.1 cm (range 162–185 cm), and the mean BMI was 24.14 kg/m^2^ (range 22.37–26.07 kg/m^2^). As shown in Table 1, there were no significant differences in the patients’ characteristics between the two groups. The median times between injury and operation were similar (1.5 weeks) between the two groups.

Table 2 shows the preoperative clinical data for the patients of the two groups. The mean anterior tibial translation was 10.57 ± 2.66 mm. The mean side-to-side anterior laxity was 3.72 ± 1.74 mm. The mean IKDC score was 49.17 ± 7.6, the mean Lysholm Knee Score was 56.56 ± 4.5, and the mean Tegner Activity Scale Score was 5.02 ± 0.74. There were no significant differences in the baseline clinical data among the patients of the two groups (all *p* > 0.05).

**Figure 1:**
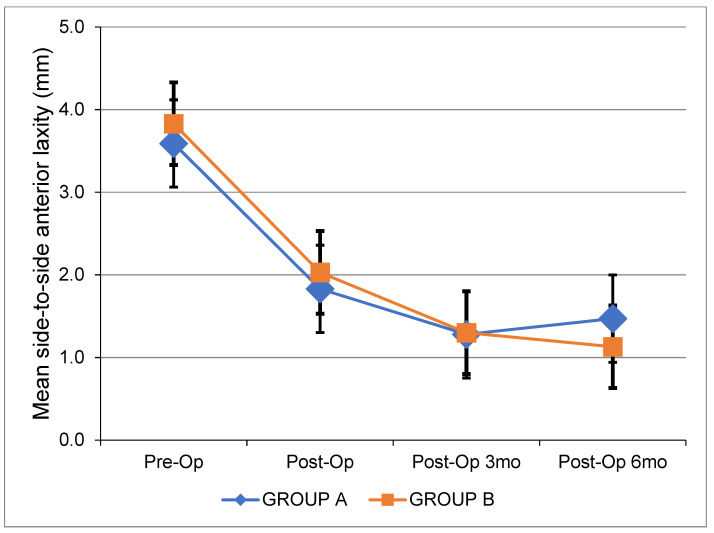
Mean side-to-side anterior laxity (mm) over time for each group. Pre-Op: Preoperatively. Post-Op: Postoperatively. mo: Months.

**Figure 2:**
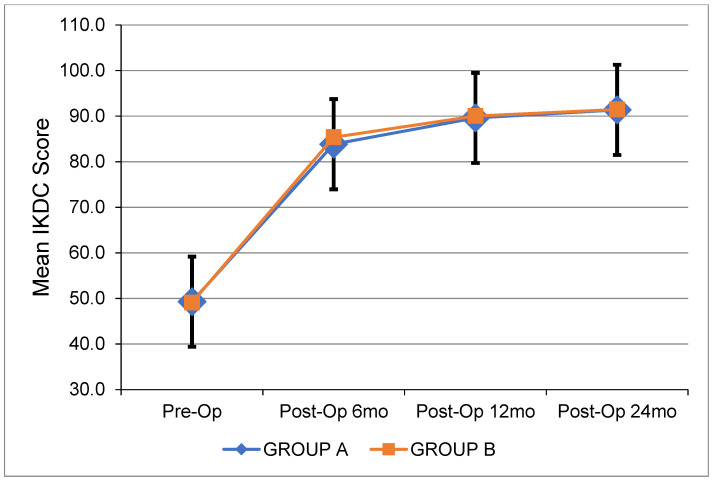
Mean IKDC score over time for each group. Pre-Op: Preoperatively. Post-Op: Postoperatively. mo: Months.

**Figure 3:**
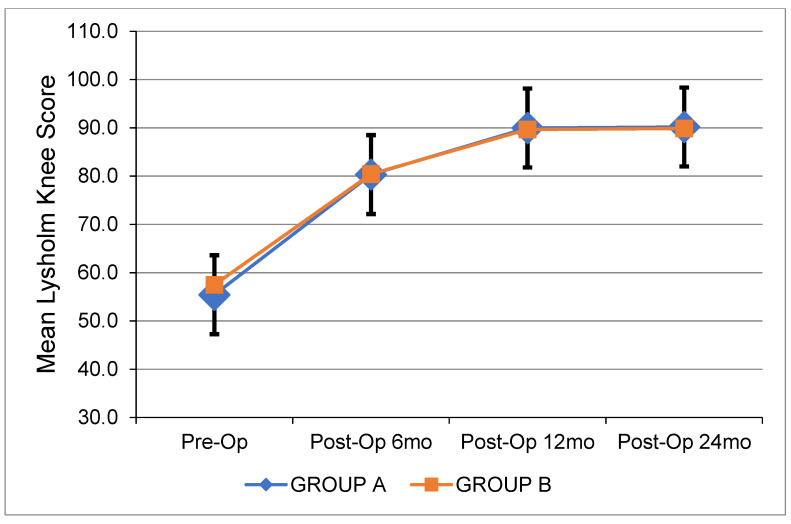
Mean Lysholm Knee Score over time for each group. Pre-Op: Preoperatively. Post-Op: Postoperatively. mo: Months.

**Figure 4:**
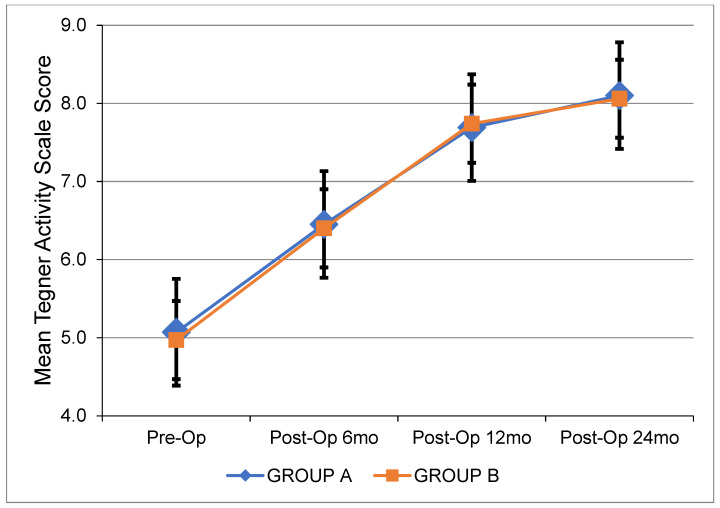
Mean Tegner Activity Scale Score over time for each group. Pre-Op: Preoperatively. Post-Op: Postoperatively. mo: Months.

Table 3 depicts all pre- and postoperative clinical data. Figure 1 shows the changes in side-to-side anterior laxity at 0, 3, and 6 months after ACL reconstruction. Figure 2, Figure 3 and Figure 4 show the change in the IKDC score, the Lysholm Knee Score, and the Tegner Activity Scale score at 6, 12, and 24 months after the operation. There were no significant differences between the two groups. The results of the pivot shift test were negative in all cases for both groups upon the respective 2-year follow-ups. The median width of the femoral and tibial tunnels was similar between the two groups (*p* = 0.172 and *p* = 0.450, respectively). No graft failures were reported at the 2-year follow-up.

## 4. Discussion

The present study compared the clinical outcomes of applying manual tension and using a tensioner device during graft fixation upon performing ACL reconstruction. The most important finding was that manual or device-assisted graft tensioning did not result in any statistically significant differences concerning the postoperative objective anteroposterior knee laxity, the IKDC score, the Lysholm knee score, and the Tegner Activity Scale score after a 2-year follow-up. No cases of graft laxity or knee instability were detected in either group.

The findings of this study agree with those reported in a randomized controlled trial by Grunau et al., who found no differences in anterior knee laxity, IKDC score, and quality of life between the manual tensioning of single-bundle hamstring ACL grafts and device-assisted tensioning among 127 ACL reconstructions [15]. On the contrary, a similar comparative study by Naik et al. reported that the Lysholm knee score after ACL reconstruction is higher when in vivo calibrated 80 N tension was applied with the use of a tensiometer during tibial attachment sparing quadruple hamstring graft fixation compared to traditional manual tensioning. However, no differences in anterior tibial translation were observed [20]. In a recent prospective study including 33 double-bundle ACL reconstructions with semitendinosus tendon autograft, Mae et al. reported that anterior knee laxity was higher when using the tensioning boot technique in comparison to the manual tensioning technique. As no clinical scores were recorded, the impact on the functional outcome was unknown [21].

The clinical outcome of arthroscopically assisted ACL reconstruction is affected by the initial graft tension at a certain degree of knee flexion [22]. Under-tensioning the graft may lead to postoperative knee joint laxity, resulting in post-traumatic osteoarthritis [23,24]. Over-tensioning of the graft may compromise the revascularization of the graft, leading to the myxoid degeneration of the graft and failure [25]. Cadaveric studies have found that increased graft tension decreases the degree of anterior translation of the tibia underneath the femur [5,26,27,28]. On the other hand, in case of graft over-tensioning, the femur may translate anteriorly onto the tibia, resulting in cartilage damage, meniscus injury, and joint stiffness [25]. Factors affecting ACL graft tension include the width and length of the graft, the type of graft, aspects regarding the preconditioning of the graft, tunnel sizes, and proper tunnel positioning [29]. To reduce variability in this study, the same surgeon performed all operations, including graft tensioning and the patients’ evaluations. The type of graft and the postoperative rehabilitation protocol were the same for all patients. There were no significant differences in the graft’s width between the two groups.

The ideal tension of an ACL graft is still a matter of controversy. In a randomized controlled trial, the application of 90 N of tension lead to a better clinical outcome in patellar tendon grafts compared to 45 N of tension [8]. Biomechanical studies using hamstring grafts found that 80 N of tension was correlated with higher postoperative knee stability compared to 40 N or 20 N [30]. Any further increase in graft tension was not beneficial [31]. After the manual pulling of the graft, the tension is transferred solely to the graft, leading to a topical creep phenomenon. Therefore, as the femur–graft–tibia complex undergoes load relaxation again, the tension may decrease after final graft fixation at the tibia [32]. In a recent cadaveric study, an initial extra-articular tensioning force of more than 80 N was associated with a significantly larger intra-articular graft force [33]. In another recent cadaveric study by Nishizawa et al., the authors observed that the intra-articular tension on the graft is significantly higher than the graft tension measured on an extra-articular device [34]. Recently, differences in the angle of tibial fixation (0° or 30°) were proved to have no significant influence on the clinical outcomes of ACL reconstruction [35]. Further studies are required to clarify if the tension applied during graft fixation is associated with intra-articular graft tension.

The technique of applying manual tension is widely preferred while performing graft fixation in ACL reconstruction as it is a simple method that is easy to perform. Some surgeons prefer to fix the graft at a certain level of tension using a manual tensioner [14,32]. However, manual tensioning has been associated with a greater graft failure rate than device-assisted tensioning (8.9% vs. 4.3%) [17]. This finding was not confirmed in the present study, as no graft failure was observed, which may be attributed to the short postoperative follow-up period. The main advantage of device-assisted tensioning is the reproducibility of the measured tension on the graft; however, the impact on clinical outcome is still an unanswered question. As manual techniques produce a variable level of graft tension ranging from 20 to 80 N, several studies support the use of a tensiometer to apply a constant and calibrated degree of tension over the graft [36]. Modern techniques have incorporated boot tensioning to free up both of a surgeon’s hands during tibial fixation [21].

The present study has certain limitations. Firstly, the selection of patients was not randomized; however, the baseline demographic and clinical data were not different among the patients of the two groups. Secondly, we supposed that the tension applied manually was similar in all cases in Group A, but we did not repeat the measurement for all patients. According to Cunningham et al., most surgeons apply a “sufficient magnitude” of initial tension, ranging from 40 to 90 N, to a graft at full extension or a slightly flexed position [37]. Lastly, MRI did not demonstrate an accurate positioning of the tibial and femoral tunnels for each patient. However, the same technique was applied to both groups. Despite these limitations, the sample size of the study was quite sufficient in comparison to similar studies. Patients’ evaluations were performed based on an arthrometer and clinical scores as we chose to rely on objective observations and not subjective data such as pain, range of motion, and clinical signs. These things considered, we believe that this study may contribute to the existing knowledge concerning the optimal fixation technique for an ACL graft.

## 5. Conclusions

No significant difference was found regarding postoperative anterior knee laxity, clinical outcomes, activity level, and patient satisfaction between the application of manual tension and the use of the ConMed Linvatec SE™ Graft Tensioning System during graft fixation while performing an anatomic single-bundle ACL reconstruction with a quadruple hamstring tendon graft. Further high-quality clinical studies are required to elucidate whether device-assisted tension is superior to manual tension.

## Figures and Tables

**Table 1:** Demographic characteristics of the study population. N: Number of patients. SD: Standard Deviation.

	Group A	Group Β	Total	*p*-Value
	N (%)	N (%)	N (%)	
**Gender**				0.226
Men	25 (86.2)	25 (71.4)	50 (78.1)	
Women	4 (13.8)	10 (28.6)	14 (21.9)	
	**Mean (SD)**	**Mean (SD)**	**Mean (SD)**	***p*-Value**
**Age (years)**	28.7 (9.4)	29.9 (9.5)	29.3 (9.4)	0.680
**Weight (kg)**	72.4 (7.0)	72.4 (5.3)	72.4 (6.1)	0.991
**Height (cm)**	173.2 (7.0)	173.1 (6.2)	173.1 (6.5)	0.945
**ΒΜΙ (kg/m^2^)**	24.1 (1.2)	24.2 (1.1)	24.1 (1.1)	0.819

**Table 2:** Preoperative clinical data regarding the study population. N: Number of patients. SD: Standard Deviation.

	Group A	Group Β	Total	
	Mean (SD)	Mean (SD)	Mean (SD)	*p*-Value
**Anterior laxity (mm)**	10.2 (2.9)	10.8 (2.5)	10.6 (2.7)	0.378
**Side-to-side anterior laxity (mm)**	3.6 (1.6)	3.8 (1.9)	3.7 (1.7)	0.576
**IKDC**	49.3 (7.5)	49.1 (7.9)	49.2 (7.6)	0.922
**LYSHOLM knee score**	55.5 (4.8)	57.5 (4.1)	56.6 (4.5)	0.076
**TEGNER activity scale score**	5.1 (0.7)	5.0 (0.8)	5.0 (0.7)	0.598

**Table 3:** Preoperative and postoperative clinical data between the 2 groups. SD: Standard Deviation. Pre-op: Preoperative score. Post-op: Postoperative score. GA: General Anesthesia. mo: Months.

	Group A	Group Β	
	Mean (SD)	Mean (SD)	*p*-Value
**Side-to-side anterior laxity (mm)**			
Pre-op	3.6 (1.6)	3.8 (1.9)	0.576
Under GA	4.5 (1.8)	4.2 (2.0)	0.523
Post-op	1.8 (1.3)	2.0 (1.7)	0.896
Post-op 3 mo	1.3 (0.9)	1.3 (1.1)	0.983
Post-op 6 mo	1.5 (1.2)	1.1 (1.0)	0.323
**IΚDC**			
Pre-op	49.3 (7.5)	49.1 (7.9)	0.922
Post-op 6 mo	83.9 (4.3)	85.4 (3.6)	0.132
Post-op 12 mo	89.6 (2.5)	90.0 (2.5)	0.517
Post-op 24 mo	91.4 (3.0)	91.5 (2.7)	0.915
**LYSHOLM knee score**			
Pre-op	55.5 (4.8)	57.5 (4.1)	0.076
Post-op 6 mo	80.3 (5.2)	80.4 (5.4)	0.930
Post-op 12 mo	90.0 (3.6)	89.7 (4.0)	0.605
Post-op 24 mo	90.2 (3.7)	89.9 (4.1)	0.614
**TEGNER activity scale score**			
Pre-op	5.1 (0.7)	5.0 (0.8)	0.514
Post-op 6 mo	6.5 (1.0)	6.4 (1.1)	0.856
Post-op 12 mo	7.7 (1.1)	7.7 (0.9)	0.938
Post-op 24 mo	8.1 (1.1)	8.1 (0.8)	0.813

## Data Availability

The data presented in this study are available on request from the corresponding author.
